# The Curcumin Analogue 1,5-Bis(2-hydroxyphenyl)-1,4-pentadiene-3-one Induces Apoptosis and Downregulates E6 and E7 Oncogene Expression in HPV16 and HPV18-Infected Cervical Cancer Cells

**DOI:** 10.3390/molecules200711830

**Published:** 2015-06-29

**Authors:** Felicia Paulraj, Faridah Abas, Nordin H. Lajis, Iekhsan Othman, Sharifah Syed Hassan, Rakesh Naidu

**Affiliations:** 1 Jeffrey Cheah School of Medicine & Health Sciences, Monash University Malaysia, Jalan Lagoon Selatan, Bandar Sunway 47500, Selangor, Malaysia; E-Mails: fsimone@gmail.com (F.P.); iekhsan.othman@monash.edu (I.O.); sharifah.syedhassan@monash.edu (S.S.H.); 2 Laboratory of Natural Products, Faculty of Science, Universiti Putra Malaysia, UPM Serdang 43400, Selangor, Malaysia; E-Mails: faridah@food.upm.edu.my (F.A.); nordinlajis@gmail.com (N.H.L.); 3 Department of Food Science, Faculty of Food Science and Technology, Universiti Putra Malaysia, UPM Serdang 43400, Selangor, Malaysia

**Keywords:** curcumin analogue, diarylpentanoid, cervical cancer, cytotoxicity, apoptosis, oncogene, E6, E7, HeLa cells, CaSki cells

## Abstract

In an effort to study curcumin analogues as an alternative to improve the therapeutic efficacy of curcumin, we screened the cytotoxic potential of four diarylpentanoids using the HeLa and CaSki cervical cancer cell lines. Determination of their EC_50_ values indicated relatively higher potency of 1,5-bis(2-hydroxyphenyl)-1,4-pentadiene-3-one (**MS17**, 1.03 ± 0.5 μM; 2.6 ± 0.9 μM) and 1,5-bis(4-hydroxy-3-methoxyphenyl)-1,4-pentadiene-3-one (**MS13**, 2.8 ± 0.4; 6.7 ± 2.4 μM) in CaSki and HeLa, respectively, with significantly greater growth inhibition at 48 and 72 h of treatment compared to the other analogues or curcumin. Based on cytotoxic and anti-proliferative activity, **MS17** was selected for comprehensive apoptotic studies. At 24 h of treatment, fluorescence microscopy detected that **MS17**-exposed cells exhibited significant morphological changes consistent with apoptosis, corroborated by an increase in nucleosomal enrichment due to DNA fragmentation in HeLa and CaSki cells and activation of caspase-3 activity in CaSki cells. Quantitative real-time PCR also detected significant down-regulation of HPV18- and HPV16-associated E6 and E7 oncogene expression following treatment. The overall data suggests that **MS17** treatment has cytotoxic, anti-proliferative and apoptosis-inducing potential in HPV-positive cervical cancer cells. Furthermore, its role in down-regulation of HPV-associated oncogenes responsible for cancer progression merits further investigation into its chemotherapeutic role for cervical cancer.

## 1. Introduction

Cervical cancer currently ranks as the second most common female cancer in the world affecting women between the ages of 15 to 44 years of age [1,2] and is responsible for the second highest cause for female cancer mortality worldwide [2]. Cervical cancer is attributed to specific human papillomavirus infections, with at least 71% of invasive cervical cancer caused by HPV high risk types 16 and 18 [3]. Upon infection, the circular viral genome often becomes integrated into the host genome and can selectively up-regulate the expression of viral oncogenes, E6 and E7, giving these cells a selective growth advantage over cells that harbour the viral genome as a nuclear plasmid [4]. These two oncogenes are responsible for the continued immortalized state of the cervical cells.

Curcumin has been identified as the active component derived from the rhizomes of the turmeric plant (*Curcuma longa*), a perennial herb used for its yellow colour and flavour in cooking. Chemically known as diferuloylmethane (bis-α,β-unsaturated β-diketone), curcumin has been shown to have a wide variety of therapeutic effects, ranging from anti-inflammatory, antioxidative, chemopreventive and anti-metastatic [5,6,7,8]. Besides its anti-metastatic and anti-proliferative effects on cancer cell growth [9], research on the signalling pathways that curcumin treatment targets, shows that it potently acts on major intracellular components involved in cancer. Curcumin affects critical genes such as NF-κβ, STAT, cyclins, MMPs, VEGF and caspases which are involved in key processes such as inflammation, genomic modulations, cell invasion and cell death pathways as reviewed by [7,8,9]. Specific reports have also discussed the role of curcumin in its antitumor activity in cervical cancer. Curcumin has shown selective cytotoxicity for HPV16- and HPV18-infected cells and induces apoptosis in cervical cancer cells by causing nuclear fragmentation, down-regulation of NF-κβ [10] and causes downregulation of E6 and E7 oncogenes and tissue specific gene expression of HPV [10,11,12].

While animal studies and clinical trials indicate that curcumin has a good safety index [13,14,15,16,17,18], the poor bioavailability of curcumin [19,20,21,22] has been highlighted as a critical issue to resolve before it can be considered as an effective therapeutic agent. Research conducted to overcome the problems with the bioavailability of curcumin includes the development of curcumin formulated with adjuvants, encapsulated by nanoparticles and liposomes or complexed with micelles and phospholipid complexes [7,23]. Recently studies have also reported success in combinatorial strategies coupling curcumin with other treatments that show synergistic effects in sensitizing resistant cells to drugs (reviewed in [24]) and suppressing cell growth by inducing apoptosis [25] and causing cell cycle arrest [26].

In addition to these studies, the use of synthetic curcumin analogues is also being studied as an option to overcome the limitations of curcumin while retaining its safety and efficacy. Structural modification studies elucidated that while specific substitutions at the aromatic rings [27,28] and heterocyclic linkers [28] enhanced the efficacy of the analogue, the 3-oxo-1, 4-pentadiene analogue was a basis for cytotoxic induction. Studies have reported that diarylpentanoids, a group of curcumin type compounds with a 5-carbon linker between its aryl rings, displayed greater growth inhibitory activity than curcumin and other 7-carbon curcuminoids [27].

The evidence for the anticancer therapeutic potential of diarylpentanoids is found in the results of numerous studies that show that diarylpentanoids induce growth suppressive effects in a wide range of cancer cell lines [29,30,31,32,33,34,35,36,37,38,39,40]. It is clear that diarylpentanoids have potential anticancer properties and to our knowledge, the study concerning the anti-carcinogenic potential of diarylpentanoids in cervical cancer is limited and merits further investigation.

Hence, it would be crucial to determine whether diarylpentanoids displayed anticancer activity by assessing its cytotoxicity, anti-proliferative activity and apoptosis-inducing potential when treated on cervical cancer cells, specifically HeLa and CaSki. Both cell lines are commonly used in *in vitro* cervical cancer research, and contain the high risk HPV types 18 and 16 viral genomes respectively. As seven out of ten cases of invasive cervical cancers are due to infection by these high risk subtypes, the use of these cell lines in the study is particularly relevant [2]. Furthermore, as HPV oncogenes play a crucial role in the progression of cervical cancer, the investigation was extended to include the study of the prospective role of the selected diarylpentanoid in inhibiting the expression of E6 and E7 oncogenes in HPV16 and HPV18-infected cervical cancer cells.

The aim of this study was to determine the cytotoxic, anti-proliferative and apoptotic activity of selected diarylpentanoid treatment on HPV-infected human cervical cancer cells as well as to study its effects on HPV-associated oncogene expression. Preliminary screening of 29 synthetic symmetrical diarylpentanoids was used to determine the potential cytotoxicity of these compounds on HeLa and CaSki cell growth. The selection process for candidate diarylpentanoids for in-depth studies prioritized compounds that dissolved well in dimethylsulfoxide (DMSO), were not strongly coloured (so as not to confound results from the colorimetric assay) and exhibited dose-dependent growth inhibitory effects compared to its untreated control. Based on these criteria, four compounds, 1,5-bis(4-hydroxy-3-methoxyphenyl)-1,4-pentadiene-3-one (**MS13**), 1,5-bis(2-hydroxyphenyl)-1,4-pentadiene-3-one (**MS17**), 1,5-bis(3-fluorophenyl)-1,4-pentadiene-3-one (**MS40E**) and 2,6-bis(3-fluorobenzylidene)cyclohexanone (**MS49**) were selected for further investigation. These four analogues were previously shown to display significant anti-proliferative activity and apoptotic properties when treated on androgen-independent human prostate cancer cells [41]. Its effects on HPV-infected human cervical cancer cells, however, are currently unknown.

## 2. Results and Discussion

### 2.1. Screening and Cytotoxicity of Diarylpentanoids

#### 2.1.1. Diarylpentanoids Induce Cytotoxic Effects on HeLa and CaSki Cell Growth

**Figure 1:**
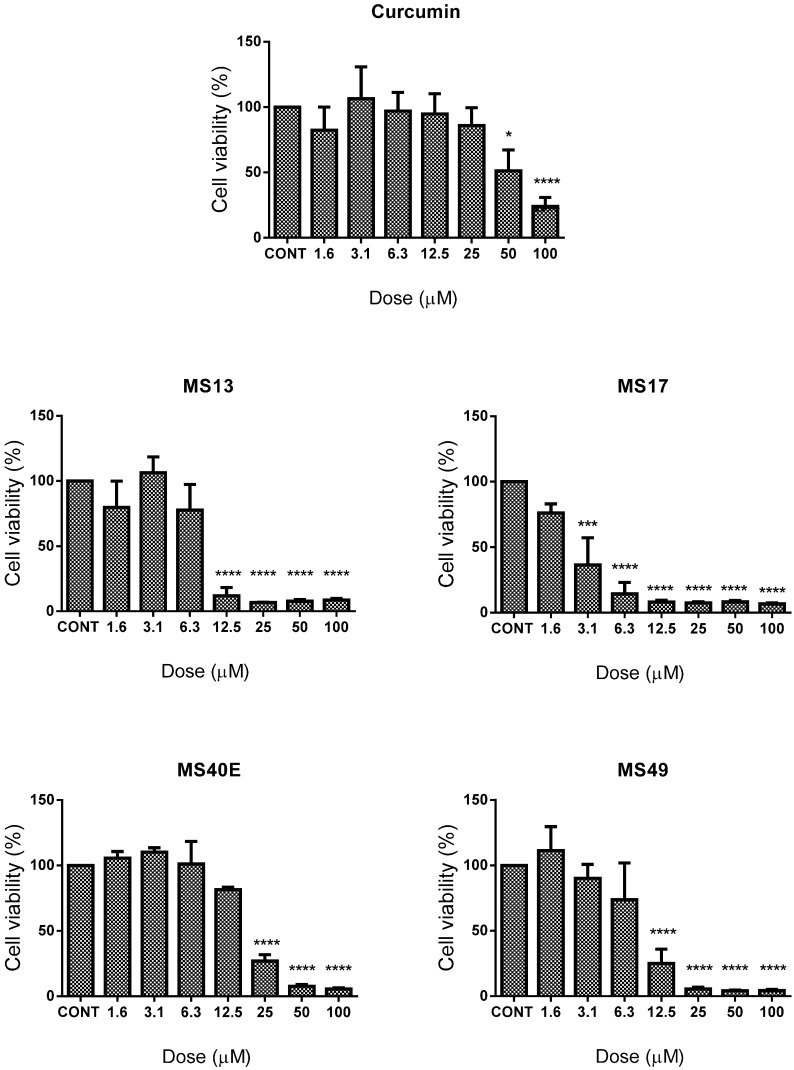
The inhibitory effects of cell viability by curcumin, **MS13**, **MS17**, **MS40E** and **MS49** in HeLa cancer cell line compared to untreated sample (CONT). Results are expressed as means of percentage cell viability and comparison between data sets performed using ANOVA. Experiments were performed in triplicates and results compared between three independent experiments. Asterisks indicate statistically significant (* for *p <* 0.05, *** for *p <* 0.001, **** for *p <* 0.0001) differences between the means of values obtained with treated *vs.* untreated cells. Error bars depict mean ± SEM.

**Figure 2:**
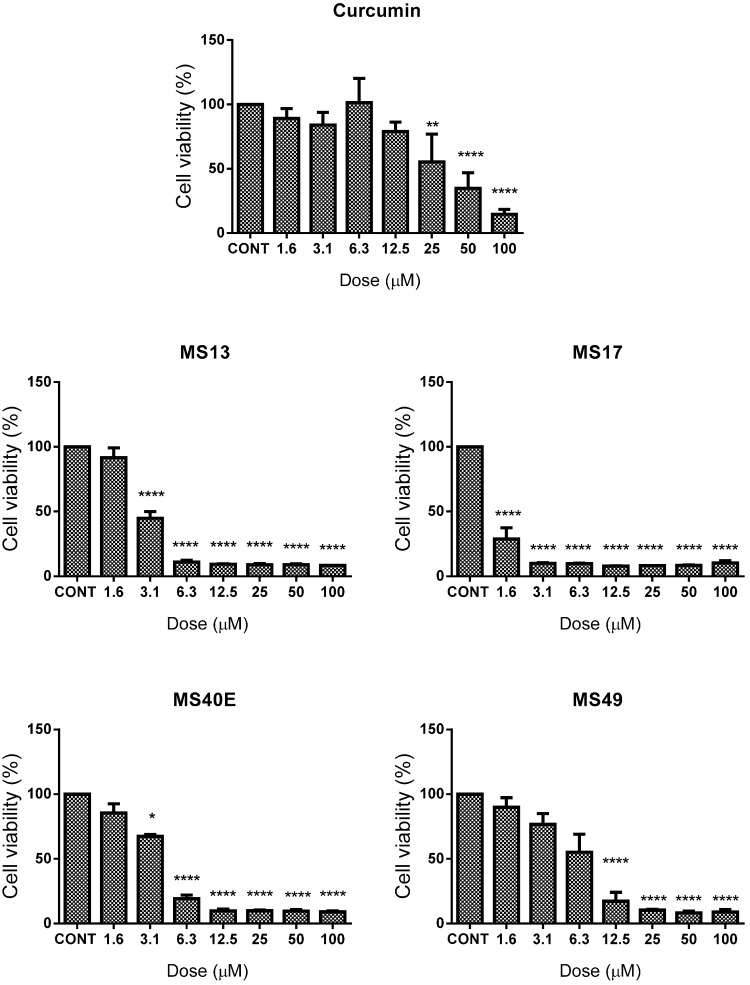
The inhibitory effects of cell viability by curcumin, **MS13**, **MS17**, **MS40E** and **MS49** in CaSki cancer cell line compared to untreated sample (CONT). Results are expressed as means of percentage cell viability and comparison between data sets performed using ANOVA. Experiments were performed in triplicates and results compared between three independent experiments. Asterisks indicate statistically significant (* for *p <* 0.05, ** for *p* < 0.01, **** for *p <* 0.0001) differences between the means of values obtained with treated *vs.* untreated cells. Error bars depict mean ± SEM.

Between treated and non-treated HeLa cells (Figure 1), **MS17** showed the most significant inhibition of cell growth with cell viability decreasing to 36% from a dose as low as 3.1 μM and gradually decreasing to 14% at 6.3 μM and then to less than 10% cell viability from 12.5 to 100 μM. **MS13** follows closely in cytotoxicity with cell viability decreasing to approximately 12% beginning from 12.5 μM and decreasing to below 10% beyond this dose. **MS49** and **MS40E** show significant growth inhibition of approximately 75% beginning at 12.5 and 25 μM respectively. **MS17** showed more potent effects in CaSki (Figure 2) compared to HeLa cells, with significant reduction in cell viability beginning from 1.6 μM (30%) followed by 90% reduction in CaSki cell viability from 3.1 to 100 μM. **MS13** followed a similar trend by exhibiting a significant decrease in cell growth beginning from 3.1 μM (50%); dosing beyond 6.3–100 μM displayed around 10% cell growth after treatment. **MS40E** showed significant growth inhibition from 6.3 μM (80%) to100 μM (90%) but **MS49** only showed a similar effect from 12.5 μM (20% cell viability) and 25–100 μM (~10% cell viability) onwards.

Curcumin on the other hand only showed significant growth inhibition of 50% at 25 μM in CaSki; a similar effect was only observed beginning at 50 μM in HeLa cells. Cell viability data was used to assess the EC_50_ values for each compound (Table 1). **MS17** had the highest inhibitory effect on CaSki and HeLa cervical cancer cell growth (CaSki EC_50_, 1.03 μM; HeLa EC_50_, 2.6 μM), followed by **MS13** (CaSki EC_50_, 2.8 μM; HeLa EC_50_, 6.7 μM), **MS49** (CaSki EC_50_, 6.0 μM; HeLa EC_50_, 8.3 μM), **MS40E** (CaSki EC_50_, 3.5 μM; HeLa EC_50_, 15.5 μM) and curcumin (CaSki EC_50_, 15.8 μM; HeLa EC_50_, 26.7 μM).

**Table 1:** *In vitro* cytotoxicity (EC_50_, µM) of curcumin and diarylpentanoids against HeLa, CaSki and MRC9 cells and their selective indices.

Compound	EC_50_ (Mean ± S.E.M.)			Selective Index	
	*HeLa*	*CaSki*	*MRC9*	*HeLa*	*CaSki*
**MS13**	6.7 ± 2.4	2.8 ± 0.4	9.7 ± 2.4	145	346
**MS17**	2.6 ± 0.9	1.03 ± 0.5	4.6 ± 1.2	177	447
**MS40E**	15.5 ± 1.9	3.5 ± 0.4	31.7 ± 3.2	204	906
**MS49**	8.3 ± 4.6	6.0 ± 2.9	16.4 ± 4.4	199	273
**Curcumin**	26.7 ± 14.3	15.8 ± 3.1	26.6 ± 3.2	100	168

Results are shown as mean ± standard deviation (S.D.) from three independent experiments.

These results indicate that, on the whole, the four diarylpentanoids displayed comparably improved dose-dependent cell growth inhibition compared to curcumin. Furthermore, it was noted that the EC_50_ values calculated for **MS17** were several fold lower in HeLa and CaSki cells compared to **MS13**, **MS40E**, **MS49** and curcumin, indicating the relatively higher potency of **MS17**. Interestingly, **MS17** also showed a cell-line specific effect as it possessed a generally lower EC_50_ value in CaSki cells compared to HeLa. All diarylpentanoids and curcumin were observed to show a similar specificity for inducing greater cytotoxicity in CaSki compared to HeLa.

Evaluation of diarylpentanoid toxicity on the non-cancerous lung fibroblast cell line, MRC9, noted that a much higher dose was required to cause MRC9 cell viability to decrease by 50% compared to the cervical cancer cell lines. The EC_50_ values of **MS13**, **MS17**, **MS40E**, **MS49** and curcumin in MRC9 were determined as 9.7 ± 2.4, 4.6 ± 1.2, 31.7 ± 3.2, 16.4 ± 4.4 and 26.6 ± 3.2, respectively. As shown previously [42], the EC_50_ values were used to calculate the selective index, SX (Table 1), a baseline used to assess the selective toxicity of the diarylpentanoids towards cancerous cells over normal cells. Not only do **MS13**, **MS17**, **MS40E** and **MS49** show SX values that exceed those of curcumin, it was noteworthy that the SX for all analogues in CaSki were several fold higher compared to HeLa.

#### 2.1.2. Diarylpentanoids Inhibit HeLa and CaSki Cell Proliferation

While the cytotoxicity of diarylpentanoid treatment was determined to be dose-dependent, we were interested in investigating whether the compounds also displayed time-dependent effects on cell proliferation rate. Cell viability was measured after treatment with diarylpentanoids for 24, 48 and 72 h. The percentage cell viability of vehicle-treated (DMSO only) cells was measured to assess MTT turnover over time in the absence of diarylpentanoid treatment. It was observed that cell viability increased significantly between 24 and 72 h, indicating that cell proliferation increases as a function of time. This pattern is noticeably altered by drug treatment in a time-dependent manner.

**Figure 3:**
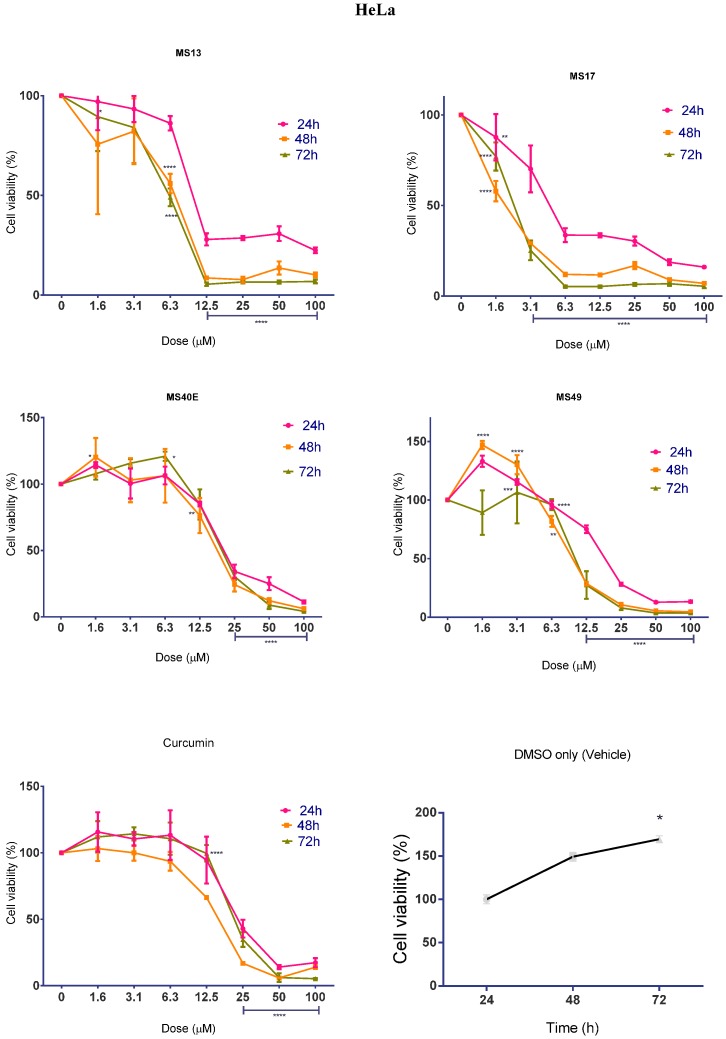
The anti-proliferative effects of **MS13**, **MS17**, **MS40E** and **MS49** on HeLa cervical cancer cells at 24, 48 and 72 h. Results are expressed as means of percentage cell viability and comparison between data sets performed using ANOVA. Vehicle-treated controls were included to assess changes in untreated cell viability over time. Experiments were performed in triplicates and results compared between three independent experiments. Asterisks indicate statistically significant (* for *p <* 0.05, ** for *p* < 0.01, *** for *p <* 0.001, **** for *p <* 0.0001) differences between the means of values obtained with treated *vs.* untreated cells. Error bars depict mean ± SEM.

When treated with **MS13** at 12.5 μM and above, HeLa cell viability (Figure 3) was significantly reduced by 72% at 24 h, and by approximately 91% and 95% at 48 and 72 h respectively. HeLa treatment with 6.3 μM onwards of **MS17** also displayed a substantial decrease of cell viability to 34% when treated for 24 h, while treatment for 48 and 72 h at the same dosage range caused cell growth to reduce to 12% and 5%, respectively. In fact, statistical comparison of the overall anti-proliferative effects of **MS13** and **MS17** on HeLa cells indicate that they were significantly higher at 48 and 72 h compared to 24 h (*p <* 0.0001). Dosing onwards from 25 μM of **MS40E** and **MS49** and 50 μM of curcumin caused significant reduction of cell growth below 50% at all three time points.

**Figure 4:**
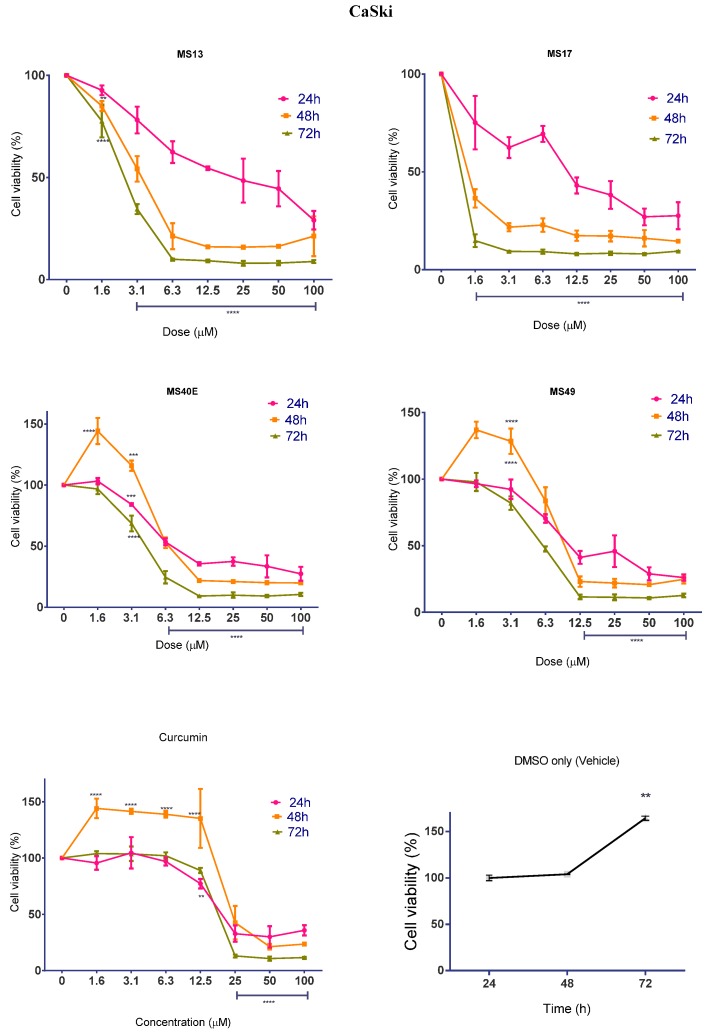
The anti-proliferative effects of **MS13**, **MS17**, **MS40E** and **MS49** in CaSki cervical cancer cells at 24, 48 and 72 h. Results are expressed as means of percentage cell viability and comparison between data sets performed using ANOVA. Vehicle-treated controls were included to assess changes in untreated cell viability over time. Experiments were performed in triplicates and results compared between three independent experiments. Asterisks indicate statistically significant (** for *p* < 0.01, *** for *p <* 0.001, **** for *p <* 0.0001) differences between the means of values obtained with treated *vs.* untreated cells. Error bars depict mean ± SEM.

In CaSki cells (Figure 4), treatment with 12.5 μM of **MS13** led to a notable decline of cell viability of approximately 50% induced by 12.5 μM at 24 h after treatment and to 21% and 9% induced from 6.3–100 μM at 48 and 72 h post-treatment. Doses from 12.5 μM onwards of **MS17** was required to reduce cell growth to 43% at 24 h, but 48 and 72 h after treatment only required a concentration range of 3.1–100 μM to reduce cell viability to 22% and 9% respectively. Similar to the effects of **MS13** and **MS17** in HeLa, comparison of the overall cell viability through time points suggested that there was a significant anti-proliferative activity that increased from 24 to 72 h (*p <* 0.0001). Treatment of CaSki cells with 12.5 μM onwards of **MS40E** and **MS49** and from 25 μM curcumin onwards caused a significant decline in cell viability at 24, 48 and 72 h.

Overall, it was noted that treatment with the diarylpentanoids and curcumin had both time- and dose-dependent anti-proliferative activity on HeLa and CaSki cells. In most cases, treatment significantly caused higher growth inhibition at 48 and 72 h compared to 24 h.

Most notably, even though **MS13** and **MS17** showed the most potent cytotoxic effects compared to **MS40E**, **MS49** and curcumin, **MS17** caused significantly greater anti-proliferative effect compared to **MS13** at 48 and 72 h after treatment.

### 2.2. Apoptotic Activity Induced by MS17

#### 2.2.1. Morphological Observation of Treated Cancer Cells Using Fluorescence Microscopy

The induction of apoptosis on HeLa and CaSki cells by **MS17** treatment was first evaluated by assessing classical morphological changes associated with apoptotic cell death by double staining with acridine orange (AO) and ethidium bromide (EB) followed by fluorescent microscopic analysis.

AO is taken up by both viable and dead or dying cells and intercalates into double stranded DNA emitting green fluorescence while EB penetrates non-viable cells and emits red fluorescence. Hence, by using previously described features [43,44], it is possible to identify between viable cells, cells undergoing early/late apoptosis and necrotic cells. Live cells with intact membranes would stain a uniform green colour while live (early) apoptotic cells would exhibit a bright green-yellow due to the chromatin condensation that occurs in early apoptosis which may take the form of bright beads around the nucleus. Late apoptotic cells would also show similar features of chromatin condensation but due to increased permeability of EB, would emit a bright red (or yellow-orange) fluorescence. Necrotic cells would be fully permeable to AO and EB and stain uniformly orange.

**Figure 5:**
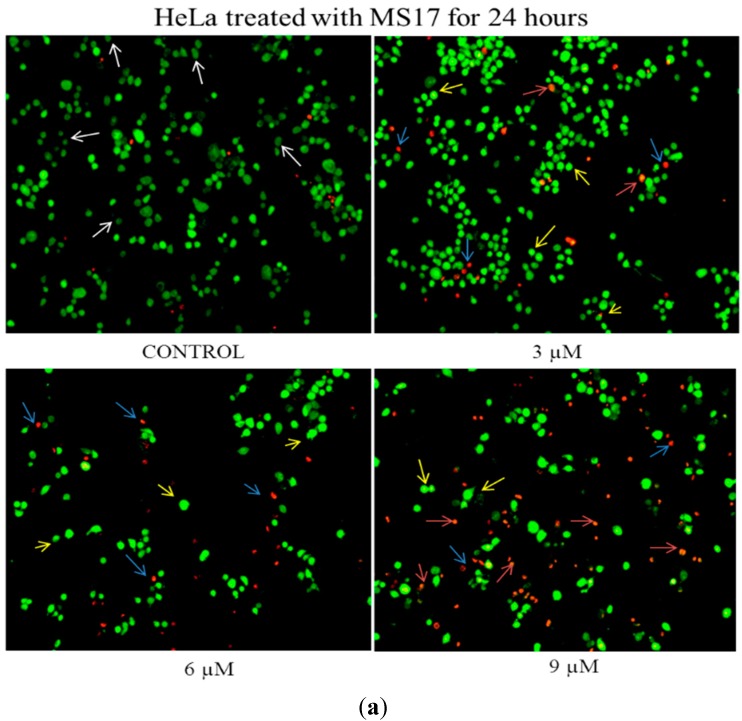

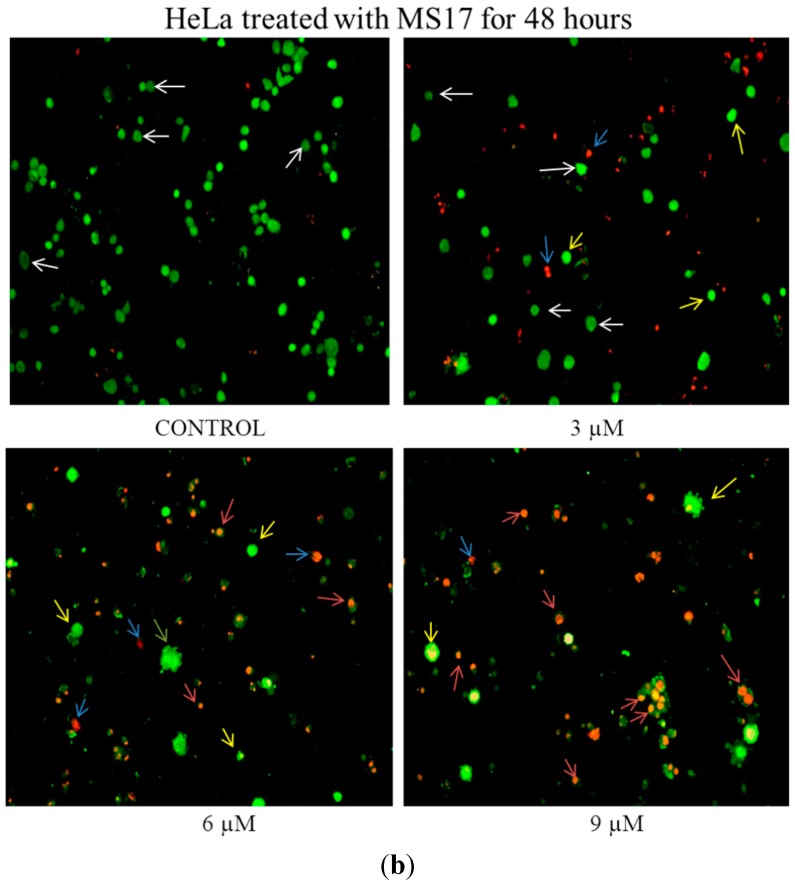
(**a**) Detection by fluorescent microscopy of acridine orange/ethidium bromide double-stained control and MS17-treated HeLa cervical cancer cells (24 h). Viable cells have uniform green nuclei with organised structure (white arrows) while early apoptotic cells would exhibit a bright green-yellow due to chromatin condensation that occurs in early apoptosis (yellow arrows). Late apoptotic cells (blue arrows) would also have bright-red (or yellow-orange) nuclei with condensed or fragmented chromatin and necrotic cells would stain uniformly orange (red arrows). Magnification 100×; (**b**) Detection by fluorescent microscopy of acridine orange/ethidium bromide double-stained control and MS17-treated HeLa cervical cancer cells (48 h). Viable cells have uniform green nuclei with organised structure (white arrows) while early apoptotic cells would exhibit a bright green-yellow due to chromatin condensation that occurs in early apoptosis (yellow arrows). Late apoptotic cells (blue arrows) would also have bright-red (or yellow-orange) nuclei with condensed or fragmented chromatin and necrotic cells would stain uniformly orange (red arrows). Magnification 100×.

**Figure 6:**
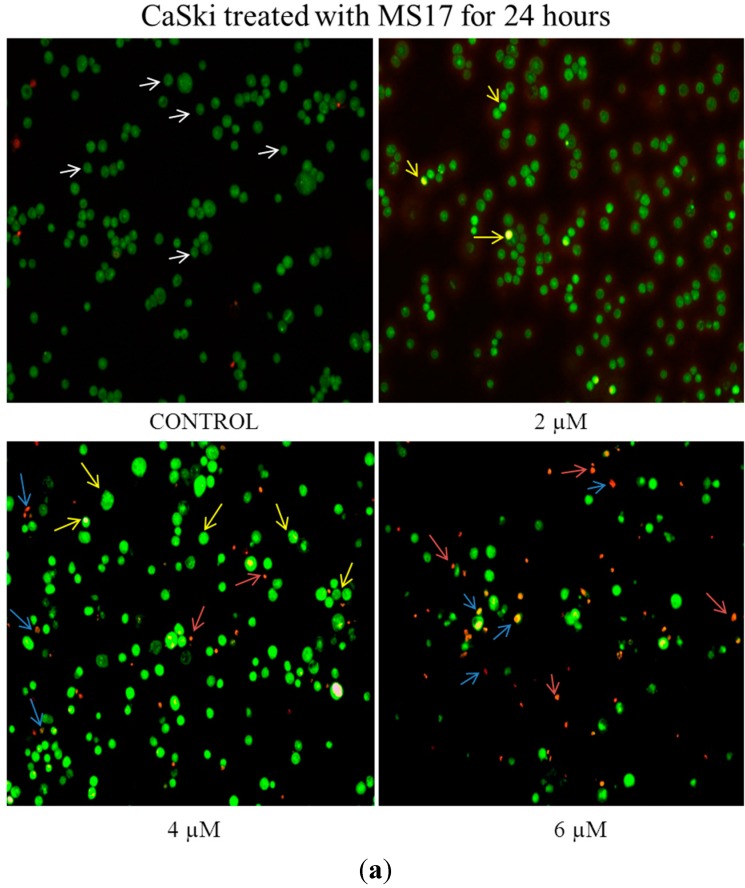

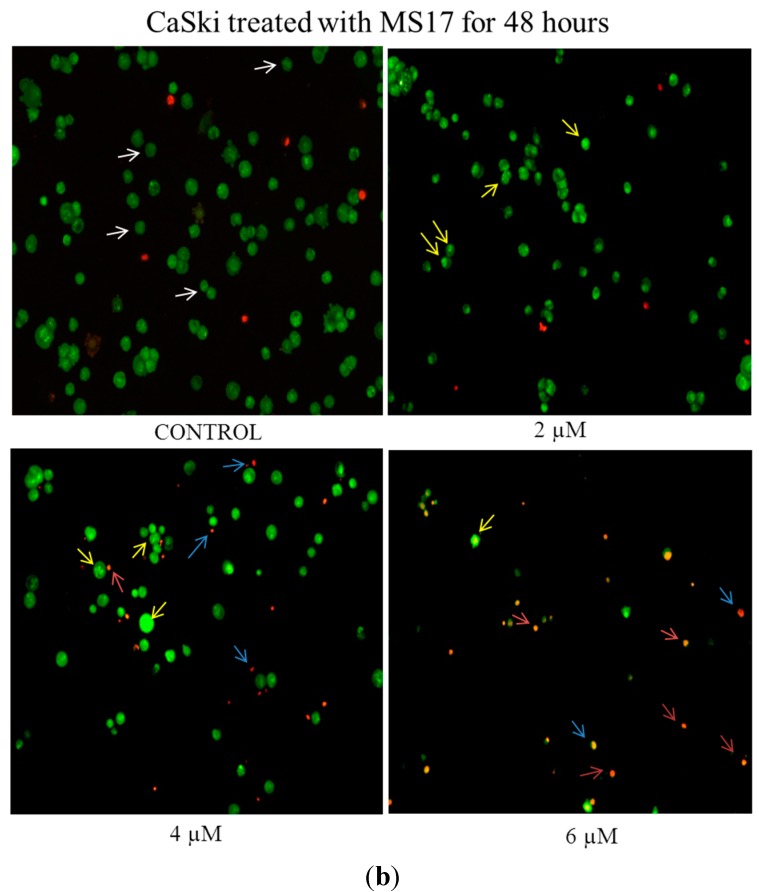
(**a**) Detection by fluorescent microscopy of acridine orange/ethidium bromide double-stained control and **MS17**-treated CaSki cervical cancer cells (24 h). Viable cells have uniform green nuclei with organised structure (white arrows) while early apoptotic cells would exhibit a bright green-yellow due to chromatin condensation that occurs in early apoptosis (yellow arrows). Late apoptotic cells (blue arrows) would also have bright-red (or yellow-orange) nuclei with condensed or fragmented chromatin and necrotic cells would stain uniformly orange (red arrows). Magnification 100×; (**b**) Detection by fluorescent microscopy of acridine orange/ethidium bromide double-stained control and MS17-treated CaSki cervical cancer cells (48 h). Viable cells have uniform green nuclei with organised structure (white arrows) while early apoptotic cells would exhibit a bright green-yellow due to chromatin condensation that occurs in early apoptosis (yellow arrows). Late apoptotic cells (blue arrows) would also have bright-red (or yellow-orange) nuclei with condensed or fragmented chromatin and necrotic cells would stain uniformly orange (red arrows). Magnification 100×.

As the concentration of **MS17** increased, the number of uniformly green viable cells (white arrows) decreased. When HeLa (Figure 5a) and CaSki (Figure 6a) cells were exposed to 3 and 2 µM respectively of **MS17** for 24 h, there was a heterogeneous population of viable and early apoptotic cells (yellow arrows) characterised by bright green or green-yellow cells with chromatin condensation.

When treated with 4 µM (CaSki) and 6 µM (HeLa), there were both early apoptotic and some late apoptotic cells that stained red (blue arrows). At 24 h after treatment with the highest dose of 6 µM (CaSki) and 9 µM (HeLa), there was an increase in the number of early apoptotic cells, late apoptotic cells and necrotic cells (red arrows) which were bright orange in appearance in CaSki and HeLa cells respectively. The gradual increase in early apoptotic cells (represented by yellow arrows) with increasing treatment concentration of **MS17** was clearly demonstrated in Figure 5a and Figure 6a. Forty eight hours after HeLa (Figure 5b) and CaSki (Figure 6b) cell treatment with 3 and 2 µM respectively, resulted in a mixed population of viable and early apoptotic cells. However in HeLa cells, there was also an observed appearance of late apoptotic cells when treated with 3 µM that was higher compared to CaSki cells treated with 2 µM. CaSki and HeLa cells treated with 4 µM and 6 µM respectively displayed increased early and late apoptotic cells and at the highest dose of 6 and 9 µM, there was a greater number of late apoptotic and necrotic HeLa and CaSki cells.

#### 2.2.2. Quantification of Apoptotic and Necrotic Cell Percentage

**Figure 7:**
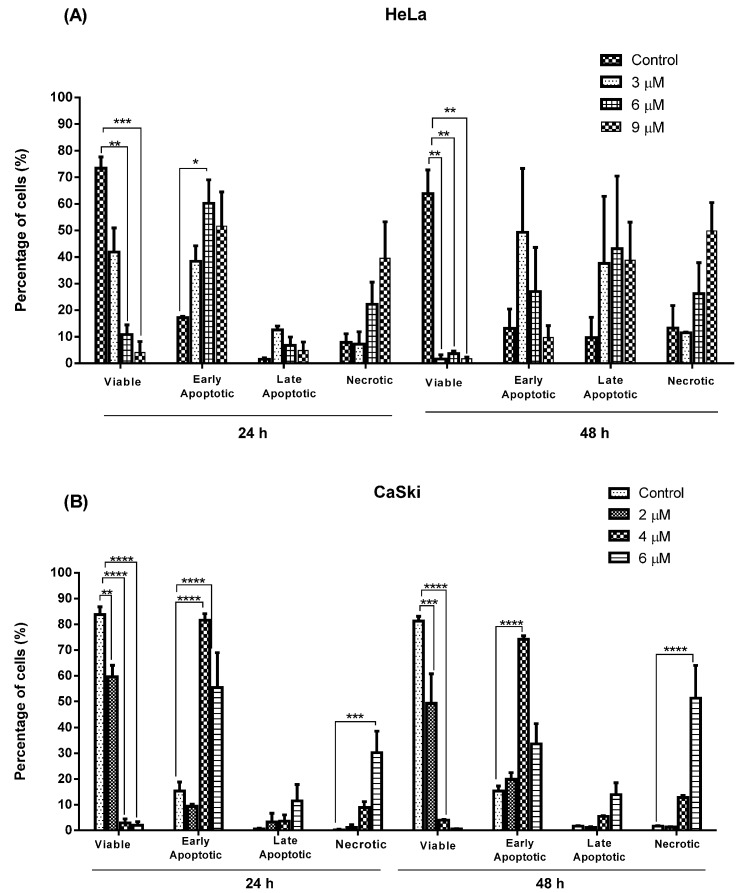
Percentage of viable, apoptotic (early and late) and necrotic cells in (**A**) HeLa and (**B**) CaSki cells treated with **MS17** for 24 and 48 h. Treated and non-treated cells were double stained and a minimum of 200 cells were counted per sample and the percentage of cells from each population (viable, live apoptotic, dead apoptotic, necrotic) was calculated. Experiments were performed in triplicates and results compared between two independent experiments. Results are expressed as means of percentage, with error bars depicting mean ± SEM. Comparison between data sets were performed using ANOVA. Asterisks indicate statistically significant (* for *p <* 0.05, ** for *p* < 0.01, *** for *p <* 0.001, **** for *p <* 0.0001) differences between data sets for each treatment dose.

In treated HeLa and CaSki cells (Figure 7), viable cells significantly decreased at 24 h in a dose-dependent manner from approximately 75% and 85% respectively to less than 10% and 5%. At 48 h after treatment, there was a reduction in the viability of HeLa and CaSki cells from 65% and 85% respectively to less than 5% with increasing treatment dose. At 24 h after treatment with **MS17**, HeLa cells displayed an increase in early apoptotic cells at 3 µM (40%), 6 µM (60%) and 9 µM (50%). After 48 h treatment, 50% of cells were maintained in early apoptosis induced by 3 µM but higher doses of 6 and 9 µM exhibited a decrease (30% and 10% respectively) in early apoptosis.

The percentage of late apoptotic cells at 24 h was relatively low at all treatment doses (≤10%) but increased to approximately 40%–45% at 48 h. Necrotic cell percentage was maintained at 3 µM (approximately 10%) and 6 µM (approximately 25%) throughout the treatment period but increased at a dosing concentration of 9 µM to 40% and 50% at 24 and 48 h respectively.

Percentage of CaSki cells undergoing early apoptosis was relatively low at 24 h (10%) and 48 h (20%) when dosed at 2 µM but significantly increased to 85% at 24 h and 75% at 48 h as treatment dose increased to 4 µM. This was followed by a reduction in early apoptotic cells when treatment was increased to 6 µM at 24 h (55%) and 48 h (30%). Unlike HeLa cells, treated CaSki cells showed a relatively low percentage of late apoptotic cells (approximately ≤10%–15%) throughout the treatment period. Treatment dose of 2 and 4 µM maintained a low percentage of necrotic cells (~10%) at 24 and 48 h but showed a significant increase to 30% and 50% respectively when treated at 6 µM.

In summary, **MS17** appears to induce early apoptosis at 24 h after treatment of CaSki cells in a time- and dose-dependent manner. The percentage of necrotic cells at this time point is also relatively low. Results from the quantification of apoptotic and necrotic cells correspond to the cell morphology pattern observed in Figure 5 and Figure 6.

#### 2.2.3. MS17 Increases Caspase-3 Activity

**Figure 8:**
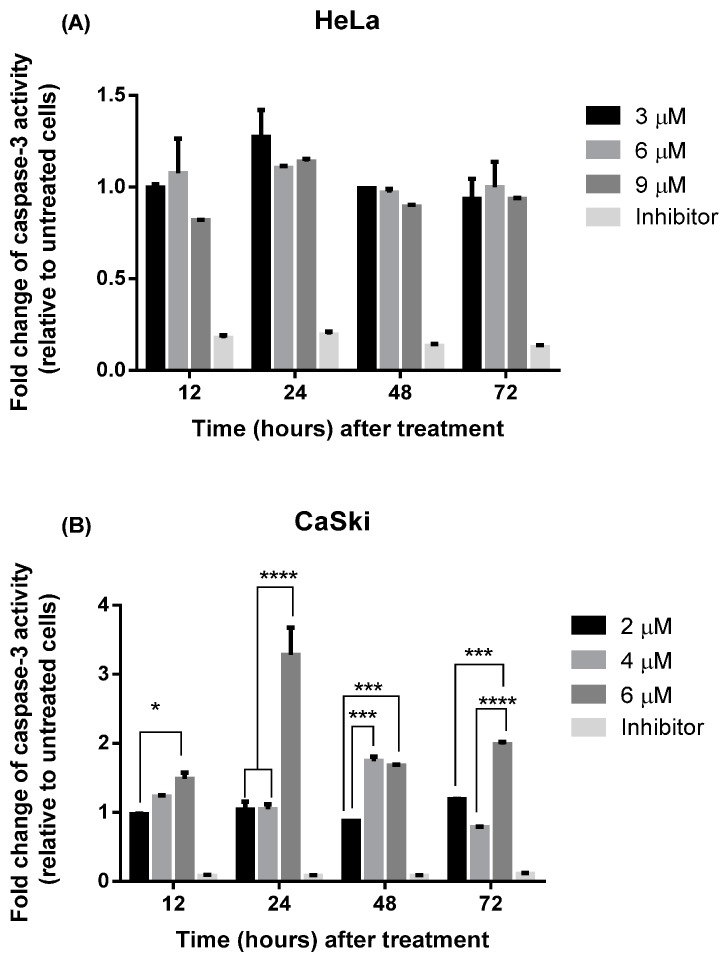
Relative caspase-3 activity in (**A**) HeLa and (**B**) CaSki cells treated with MS17 at different time points. Experiments were performed in duplicates and results compared between two independent experiments. Results are expressed as the ratio of means of caspase-3 activity of treated samples over untreated samples and comparison between data sets performed using ANOVA. Asterisks indicate statistically significant (* for *p <* 0.05, *** for *p <* 0.001 and **** for *p <* 0.0001) differences between data sets for each treatment dose. Error bars depict mean ± SEM.

The caspase-3 quantification assay (Figure 8) was performed to address whether treatment of the cervical cancer cells with **MS17** causes increased caspase-3 activity.

CaSki cells showed low caspase-3 activity when treated with 2 μM and increased activity was noted at 48 h after treatment with 4 μM. However there was a significant threefold increase in relative caspase-3 activity observed 24 h after treatment with 6 μM. In HeLa cells however, there were no significant differences observed in caspase-3 activity across all time points and doses upon **MS17** treatment. The data indicates that **MS17** induces highest caspase-3 activity at 24 h following treatment with 6 μM in CaSki cells while there was no significant difference in treated HeLa cells between time points after treatment.

#### 2.2.4. MS17 Induces DNA Fragmentation

**Figure 9:**
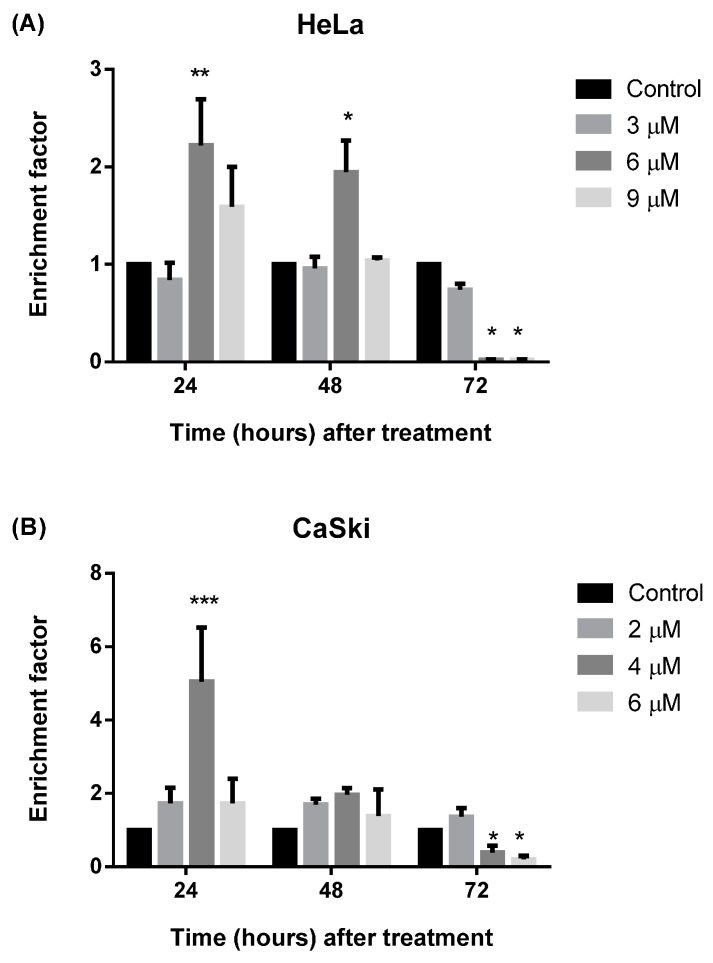
Detection of nucleosomal enrichment in the cytoplasm of (**A**) HeLa and (**B**) CaSki cells treated with different concentrations of **MS17** for 24, 48 and 72 h. Experiments were performed in duplicates and results compared between at least two independent experiments. Enrichment factor is expressed as mean ratio, with error bars depicting mean ± SEM. Comparison between data sets was performed using ANOVA. Asterisks indicate statistically significant (* for *p <* 0.05, ** for *p <* 0.01 and *** for *p <* 0.001) differences between the mean ratio of values obtained with treated *vs.* untreated cells.

A third validation assay was performed to assess whether exposure to **MS17** caused cell death via apoptosis by studying the cytoplasm of treated cells for the presence of liberated oligonucleosomes due to DNA fragmentation caused by the treatment (Figure 9).

Increased cytoplasmic nucleosomal enrichment leads to enhanced enrichment factor, which denotes apoptotic activity. Treatment with 6 μM (HeLa) and 4 μM (CaSki) caused a significant 2.2- and 5-fold increase in nucleosome enrichment in HeLa (*p <* 0.01) and CaSki (*p <* 0.001) respectively which peaked at 24 h after treatment but decreased thereafter. Treatment of HeLa cells with 6 μM at 48 h post-treatment also showed approximately two-fold increase in enrichment factor compared to the untreated cells (*p <* 0.05) but was significantly lower compared to enrichment at 24 h at the same treatment dose. There is no significant difference between doses at 48 h post-treatment for CaSki. At 72 h post-treatment there is a significant reduction of enrichment factor in HeLa cells when treated with 6 and 9 μM but no significant reduction was noted for CaSki cells.The data indicates that **MS17** induces apoptosis in HeLa and CaSki by DNA fragmentation at 24 h after treatment.

### 2.3. **MS17** Down-Regulates E6 and E7 Viral Oncogene Expression

**Figure 10:**
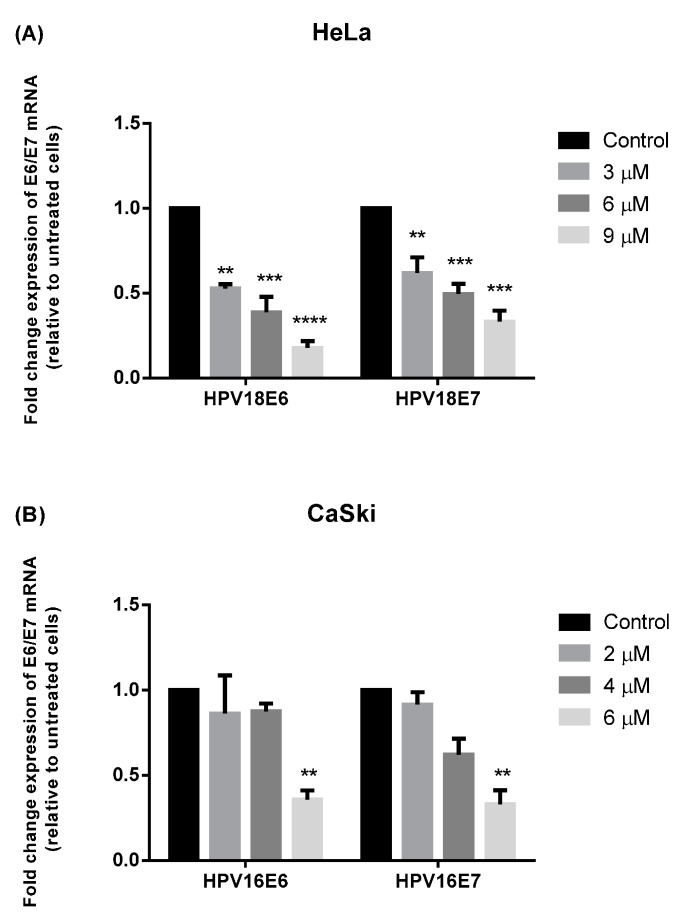
E6 and E7 viral oncogene expression of treated (**A**) HeLa and (**B**) CaSki cells. At 24 h post-treatment with **MS17**, HPV18 E6/E7 and HPV16 E6/E7 mRNA levels in HeLa and CaSki cells were measured by quantitative real time PCR and normalized to β-actin mRNA levels. Expression is reported as mean fold-change in the level of mRNA in treated cells relative to control cells. Experiments were performed in duplicates and results compared between two independent experiments. Asterisks indicate statistically significant (** for *p <* 0.01, *** for *p <* 0.001 and **** for *p <* 0.0001) differences between the mean of values obtained with treated *vs.* untreated cells. Error bars depict mean ratio ± S.E.M.

Quantitative real-time polymerase chain reaction (RT-PCR) was used to study whether treatment with **MS17** led to potential changes in oncogenic E6 and E7 mRNA expression in cervical cancer cells (Figure 10).

Expression of HPV18 E6 and E7 viral oncogene reduced significantly between approximately 1.9- to 5.6-fold and 1.6- to 3-fold, respectively, compared to untreated HeLa cells. Most notable was that treated HeLa cells showed significantly greater reduction of E6 (*p <* 0.0001) and E7 (*p <* 0.001) expression at 9 μM. Treatment on CaSki cells also exhibited a dose-dependent decrease in oncogene expression; while treatment with 2 μM and 4 μM did not show significant inhibition of E6 and E7 oncogene expression, treatment with 6 μM displayed a significant 3-fold decrease in HPV16E6 and HPV16E7 expression (*p <* 0.01) compared to untreated cells. Overall, treatment of HeLa cells with 9 μM and CaSki cells with 6 μM respectively significantly reduced E6 and E7 viral oncogene expression in HeLa and CaSki cells. Hence, our data suggests that **MS17** might be involved in the down regulation of HPV16 and HPV18 E6 and E7 oncogene expression.

### 2.4. Discussion

The present study aims to explore the potential anticancer activity of curcumin-like diarylpentanoids when treated on HPV-positive HeLa and CaSki human cervical cancer cells. Candidate compounds were shortlisted for in-depth study of their apoptotic capabilities by assessment of its cytotoxic potential compared to the parent compound, curcumin.

Following treatment of HeLa and CaSki cells with four curcumin analogues, **MS13**, **MS17**, **MS40E** and **MS49**, it was observed that each compound exerted significant dose-dependent inhibition of cell viability that was improved over curcumin. There were however notable variations in terms of potency; **MS17** showed the most cytotoxic effect on CaSki and HeLa cell viability corroborated by its relatively lower EC_50_ values, closely followed by **MS13** which showed EC_50_ values which were approximately 2.5-fold higher.

In previous investigations, Fajardo and colleagues established that curcumin analogue 27 (Ca27) which shares the same structure as **MS17** caused significant cell death of C4-2 and LNCaP prostate cancer cells at concentrations of more than 2.5 μM and 10 μM respectively [30]. They concurred with several groups in assigning the potential cytotoxic capability of the analogue to its structure, specifically the α,β-unsaturated ketone, which acts as a Michael acceptor for nucleophilic groups [45].

FLLL-11, which is identical to **MS13**, was tested in breast and prostate cancer cells and reported IC_50_ values between 0.3 and 5.7 μM which were several fold lower than curcumin [46]. Hence the literature supports the cytotoxic effects of **MS13** and **MS17** in inducing cell death in prostate and breast cancer cell lines and to the best of our knowledge, ours is the first study comparing the effects of **MS13** and **MS17** in HeLa and CaSki cervical cancer cells.

It was also observed that HeLa and CaSki demonstrated differing sensitivities to treatment with the diarylpentanoids (and curcumin); in each instance, treatment of CaSki consistently resulted in lower EC_50_ values compared to HeLa cells, and therefore suggests greater potency in CaSki compared to HeLa. Although both HeLa and CaSki are infected with high risk subtypes that cause cervical cancer, the cell lines originate from different sources. CaSki is a squamous cell carcinoma that makes up part of the epidermis while HeLa is an adenocarcinoma formed in epithelial glands. The derivation of CaSki and HeLa from different sources may explain the possible differences in their EC_50_ values in response to diarylpentanoid treatment.

Assessment of the SX values of diarylpentanoid treatment on cervical cancer cells revealed that **MS13**, **MS17**, **MS40E** and **MS49** showed selective toxicity towards HeLa and CaSki over the non-tumoral cell line, MRC9. Of note is the fact that **MS40E** showed almost 4.5-fold higher SX in CaSki which may allude to the fact CaSki cells are particularly sensitive to the effects of MS40E. On average, it was observed that all diarylpentanoids displayed greater selectivity for toxicity in CaSki cells over HeLa.

While the low EC_50_ values of diarylpentanoid treatment suggest a dose-dependent cytotoxic effect, treatment at 24, 48 and 72 h also indicated inhibition of cell proliferation rate in a time-dependent manner. Vehicle-treated control cells display proliferation over time while treatment appears to have a significant inhibitory impact on cell growth especially at later time points.

Treatment of CaSki and HeLa cells from 12.5 and 25 μM respectively of **MS40E** and **MS49**, and treatment with 25 and 50 μM onwards of curcumin, caused a notable decline in cell viability at 24, 48 and 72 h. However **MS13** and **MS17** exerted a substantial impact in anti-proliferative activity. Treatment with both diarylpentanoids induced significantly higher reduction in cell viability at 48 and 72 h compared to 24 h but **MS17** induced this effect at a dosage range that was two-fold lower (from 6.3 and 3.1 μM respectively) compared to **MS13** (from 12.5 and 6.3 μM respectively) in HeLa and CaSki cells. This suggests that the cytotoxic effects of the diarylpentanoids are largely influenced by dosage as well as treatment length.

Apoptosis has been discussed as a common mechanism employed by cytotoxic agents [47,48] to induce cell death [49]; once initiated, the apoptotic process causes DNA fragmentation and morphologic changes which include blebbing, cell shrinkage, nuclear fragmentation and chromatin condensation [50]. Having established that **MS17** induces time- and dose-dependent cytotoxic effects on HeLa and CaSki at relatively low EC_50_, a series of assays were employed to study whether key morphologic and biochemical hallmarks associated with apoptosis were present after treatment with MS17. In order to determine whether the effect of treatment was modulated by treatment dosage and/or time, cervical cancer cells were exposed to **MS17** at approximate concentrations of its respective EC_50_, 2× EC_50_ and 3× EC_50_ at various time points.

In the morphological discrimination of stained cells, it was observed that there was a heterogeneous cell population at each treatment dose, a typical property of cancer cells. Nonetheless, at specific doses, particular populations dominated over the others. It was noted that 24 h after HeLa and CaSki treatment with higher doses of **MS17** resulted in a large proportion of early apoptotic cells compared to late apoptotic cells, denoted by bright green-yellow cells with chromatin condensation, which are classic features of apoptosis. A combination of high treatment dose and prolonged exposure, particularly with 6 µM (CaSki) and 9 µM (HeLa) for 48 h, led to the appearance of many bright orange cells as well, indicating that a proportion of the cells could be induced to undergo necrosis as a method of cell death, rather than apoptosis. These results are interesting as it denotes that a higher dosage for chemotherapy may not always be more effective; necrosis has many adverse cellular consequences while apoptosis causes little tissue disruption and no inflammation [50]. This could be one of the many reasons apoptosis has been heralded as a more efficient strategy for chemotherapy rather than necrosis. Hence, morphological assessment of treated HeLa and CaSki cells suggests that **MS17** has the capability of inducing early apoptosis in a time- and dose-specific manner.

In the induction of programmed cell death, many key factors play crucial roles in mediating apoptotic response. Caspases are a family of proteinases that are constitutively present as zymogens within the cell; when cleaved, its activation in turn initiates a cascade of proteolytic cleavage of caspases. Activation of the key downstream executor, caspase-3, results in the morphological characteristics associated with apoptosis and eventually leads to DNA degradation [51]. It is therefore notable that in the present work, caspase-3 activity was noted to be highest at 24 h following treatment with 6 µM in CaSki cells. Treatment with **MS17** also induces a peak in nucleosome enrichment due to DNA fragmentation observed at 24 h in CaSki (4 µM) and HeLa (6 µM) cells. However, there were no observable significant differences of caspase-3 activity in treated HeLa cells. This was interesting to us as **MS17**-treated HeLa cells were showing features of apoptotic activation in morphological assessment and DNA fragmentation and suggests that the mechanism of apoptosis induced in HeLa cells by **MS17** may be caspase-3 independent. While HeLa typically undergoes caspase-dependent apoptosis, previous literature has shown that depending on the compound it is exposed to, HeLa cells have been shown to induce a caspase-independent apoptotic process [52].

The cytotoxic and apoptotic effects of **MS17** treatment were accompanied by decrease of E6 and E7 viral oncogene expression in HeLa and CaSki cells across doses but displayed a highly significant downregulation at 9 and 6 μM respectively, proposing that **MS17** may also be involved in interfering or associating with the cellular processes that regulate viral gene expression. This could have significant implications as E6 and E7 are crucial oncoproteins due to its interaction with tumor suppressor proteins p53 [53] and retinoblastoma (Rb) [54] respectively. Interestingly, E6 and E7 can immortalize cells independently but when present together, shows a synergistic effect that has marked efficiency in transforming activity [55]. Studies have shown that mutations in the E6 or E7 open reading frame completely abolished the tumorigenicity of cervical cancer cells suggesting that both E6 and E7 oncogenes are necessary for the malignant phenotype of HPV-positive cervical cancer cells [56,57]. E7 interacts with and degrades Rb and releases transcription factor E2F from Rb inhibition, thus stimulating cyclin genes [58], inactivate their inhibitors [59,60,61] and contributes to tumorigenesis. The efficient binding of Rb by E7 would typically lead to inhibited cell growth and induce apoptosis through a p53-dependent pathway in E7-expressing cells. Thus E6 has evolved to cause degradation of p53; as a consequence, p21 protein is not made available as a “stop” signal for cell division thus resulting in genomic instability. Furthermore, the pro-apoptotic protein, Bak, is also degraded causing resistance to apoptosis [62].

The results reported here correspond with studies where curcumin treatment of cervical cancer cells inhibits transcription of E6 and E7 oncogenes and restores the expression of tumour suppressor proteins p53 and retinoblastoma [11]. Singh *et al*., have demonstrated [63] that curcumin is able to induce apoptosis and E7 oncoprotein down-regulation in HPV-positive cervical cancer cells. Significant decline in the expression levels of E6 and E7 proteins in SiHa HPV16-positive cells treated with curcumin for 24 h [64] was noted and they concluded that loss of E6/E7 culminates in apoptotic cell death in these cervical cancer cells. Hence, down-regulation of key oncogenes in this study could be contributing to the sensitivity of the cervical cancer cells to apoptotic cell death when treated with **MS17**.

Overall, the present study demonstrates that treatment with four curcumin diarylpentanoids, **MS13**, **MS17**, **MS40E** and **MS49**, had comparably improved cell viability inhibition of HPV-positive human cervical cancer cells compared to the parent compound, curcumin. Anti-proliferative studies indicate that **MS17** treatment caused significant inhibition of cell proliferation at 48 and 72 h of treatment, compared to 24 h. The potential candidate **MS17** was chosen based on cytotoxic and anti-proliferative data and a series of downstream apoptotic assays suggest that **MS17** induces DNA fragmentation and classical morphological features of apoptosis such as chromatin condensation in HeLa and CaSki cells as well as activation of caspase-3 activity in CaSki cells. **MS17** also causes downregulation of crucial HPV oncogenes that are responsible for progression of cervical carcinoma which may also result in cell death. Future treatments of cervical cancer may also include the exploration of combinatorial strategies of **MS17** with chemotherapeutic drugs that are currently in use. On its own, curcumin has shown strong cytotoxicity on Hodgkin’s Lymphoma (HL) cells but when used in combination with cisplatin, a more potent toxic effect was noted. It was hypothesized that curcumin may be involved in cisplatin sensitization in HL cells and the synergistic effects of curcuminoids [65], including MS17 with other drugs, presents an intriguing avenue for future investigation.

To the best of our knowledge, the data in this study is a novel report describing the cytotoxic, anti-proliferative and apoptotic effects of the diarylpentanoid **MS17** in HeLa and CaSki cervical cancer cells. Nonetheless, these initial findings warrant further investigation to fully elucidate the molecular pathways involved in the mechanism of its action as well as the assessment of its effects using *in vivo* models.

## 3. Experimental Section

### 3.1. Cell Culture

HPV-infected human cervical cancer cell lines, HeLa and CaSki, and human fetal lung fibroblasts, MRC-9, were obtained from American Type Culture Collection (ATCC, Rockville, MD, USA) and maintained using appropriate media. HeLa and MRC-9 were maintained using MEM medium (Gibco, Grand Island, NY, USA), and RPMI 1640 (Gibco) was used to maintain CaSki cells. All cell lines were maintained with respective media supplemented with 10% Fetal Bovine Serum (FBS, Gibco) and penicillin (100 U/mL)/streptomycin (100 µg/mL) (Gibco). These cells were cultured at 37 °C in a humidified atmosphere with 5% CO_2_. HeLa and CaSki cells are reported to contain HPV type 18 and 16 (high-risk) viral genome sequences respectively.

### 3.2. Preparation of Curcumin Analogues

Four chemically synthesized and purified diarylpentanoids [41], namely 1,5-bis(4-hydroxy-3-methoxyphenyl)-1,4-pentadiene-3-one (**MS13**), 1,5-bis(2-hydroxyphenyl)-1,4-pentadiene-3-one (**MS17**), 1,5-bis(3-fluorophenyl)-1,4-pentadiene-3-one (**MS40E**) and 2,6-bis(3-fluorobenzylidene)cyclohexanone (**MS49**) were prepared by coupling the appropriate aromatic aldehyde with acetone, cyclopentanone and cyclohexanone under base catalyzed aldol condensation, using a ketone-aldehyde ratio of 1:2 [66]. All compounds were characterized based on analysis of their spectroscopic data and comparison of these data with those of related compounds. Curcumin was obtained from commercial sources (Sigma Aldrich, St. Louis, MA, USA). 50 mM concentrations of diarylpentanoid stock solutions were prepared in DMSO (Sigma Aldrich).

### 3.3. Cell Viability and Anti-Proliferative Assays

HeLa, CaSki and MRC9 cells were plated in triplicate wells in 96-well flat-bottomed tissue culture plates (Nunc, Roskilde, Denmark) with 10^5^ cells per mL in 100 μL culture media with working concentrations of diarylpentanoid treatment ranging from 1.6 to 100 μM. Control-treated wells contained cells incubated in media with equal volumes of DMSO (0.2%). Cells were treated for 72 h for dose-dependent cytotoxicity assays and at 24, 48 and 72 h for anti-proliferative (time-dependent) assays. In order to assess cell viability and anti-proliferative activity after diarylpentanoid treatment, the colorimetric 3-(4,5-dimethylthiazol-2-yl)-2,5-diphenyltetrazolium bromide (MTT) assay was performed as previously described with modifications [67]. The media was aspirated and cells were incubated in 100 µL complete medium with 0.5 mg/mL MTT solution for 4 h in the dark to allow metabolism of MTT by cellular mitochondrial dehydrogenases. The excess MTT was aspirated and the formazan crystals formed were dissolved by the addition of 100 µL of DMSO. The absorbance of dark blue formazan was read at 570/650 nm wavelengths using a microplate reader (Bio-Rad Benchmark Plus Microplate Spectrophotometer, Hercules, CA, USA). Triplicate absorbance values were averaged and the effect of the compounds on growth inhibition was assessed as percentage cell viability as follows:

(1) $$\text{Percentage cell viability =}\frac{\text{Absorbance of Test }-\text{ Absorbance of Blank (media only)}}{\text{Absorbance of Control }-\text{ Absorbance of Blank (media only)}}\;\text{× 100}$$

where, absorbance = optical density of sample, O.D.; control = untreated sample; test = treated sample.

An independent set of vehicle-treated (DMSO only) controls were included in the anti-proliferative assays to assess the proliferation of untreated cancer cells over 24, 48 and 72 h. As such, the cell viability at 24 h was used as a baseline to measure proliferation rate at 48 and 72 h.

(2) $$\text{Percentage cell viability = }\frac{\text{Absorbance of Control (Time) × Absorbance of Blank (media only)}}{\text{Absorbance of Control (24 h) × Absorbance of Blank (media only)}}\text{ × 100}$$

The results were analysed using GraphPad Prism version 6.00 for Windows (GraphPad Software, La Jolla, CA, USA). Using percentage cell viability Equation (1), log dose–response curves were constructed, from which the half maximal effective concentration, EC_50_ for each replicate was determined. Overall EC_50_ was determined by taking the average EC_50_ from three independent experiments. Using the EC_50_ values of the compounds obtained from cytotoxicity assays in HeLa, CaSki and MRC9 cells lines, the selectivity index (SX) values of the diarylpentanoids were calculated according to the equation below as previously established [42].

(3) $$\text{Selectivity Index (SX) =}\frac{{\text{EC}}_{50}\text{ (MRC9)}}{{\text{EC}}_{50}\text{ (Cancer cell line)}}\text{ × 100}$$

SX is used to determine whether a selected compound possesses greater selectivity for achieving therapeutic effects in cancerous cells while producing minimal toxicity on normal cells. Similar to previous practice [42,68], “100” was determined as the baseline; compounds that showed a SX value more than 100 would indicate a dosing concentration that selectively achieves higher therapeutic potential for cancerous cells over causing toxicity in non-cancerous cells; these compounds would ideally be chosen for further development. Selected diarylpentanoids were further studied in apoptotic induction assays.

### 3.4. Induction of Apoptosis with Selected Diarylpentanoids

For apoptotic assays, cancer cells were exposed to MS17 at approximate concentrations of its respective EC_50_ [CaSki, 2 μM; HeLa, 3 μM], 2× EC_50_ [CaSki, 4 μM; HeLa, 6 μM] and 3× EC_50_ [CaSki, 6 μM; HeLa, 9 μM] at various time points. Each experiment included a set of untreated control cells (Media with DMSO alone).

#### 3.4.1. Acridine Orange-Ethidium Bromide Staining for Morphological Evaluation of Apoptosis

Double staining with acridine orange and ethidium bromide and consequently fluorescent microscopic analysis is an established method by which to distinguish between viable, necrotic and apoptotic cells [43,44]. HeLa and CaSki cervical cancer cells were exposed to MS17 for 24 and 48 h at concentrations of their respective EC_50_, 2× EC_50_ and 3× EC_50_. Following treatment, the cells were pelleted, washed twice in 1× PBS and resuspended in 50 μL 1× PBS. 1 μL of dye mixture (1 part 100 μg/mL acridine orange in PBS and 1 part 100 μg/mL ethidium bromide in PBS) was added to 10 μL of cell suspension and the entire volume was placed on a clean microscope slide. Cells were examined by fluorescence microscopy using the fluorescein filter (BX41, Olympus, Melville, NY, USA) and were distinguished via the morphological aspect of chromatin condensation in stained nuclei as previously established [69]. A minimum of 200 cells were counted per sample and the percentage of cells from each population (viable, live apoptotic, dead apoptotic, necrotic) was calculated.

#### 3.4.2. Quantification of Relative Caspase-3 Activity

Caspase-3 activity was assessed using the Caspase-3 Colorimetric Assay Kit (Sigma Aldrich), following the manufacturer’s instructions. Briefly, 4 × 10^5^ cervical cancer cells were plated in 6-well tissue culture plates, left to attach for 24 h before being exposed to MS17 at concentrations of its respective EC_50_, 2× EC_50_ and 3× EC_50_ for 12, 24, 48 and 72 h. Following treatment, the cells were washed in PBS and suspended in a lysis buffer (50 mM HEPES pH 7.4, 5 mM CHAPS, 5 mM DTT) for 25 min at a concentration of 107 cells per 100 μL of buffer. Lysed cells were centrifuged at 16,000× *g*, 4 °C for 30 min. Lysate protein concentration was determined using the Cayman Chemical Protein Determination (Cayman Chemical Company, Ann Arbor, MI, USA) kit which is a colorimetric method for total protein quantification based on the Bradford method. In short, 100 μL of protein samples was diluted in 96-well plates and 100 μL of the assay reagent was added to the samples, incubated for 5 min and the absorbance measured at 595 nm. Protein concentrations were calculated from a regression line fit to a series of standard protein dilutions assays on the same plate as the unknown samples. Approximately 100 μg total protein from each sample were added to wells containing the assay buffer (20 mM HEPES, pH 7.4, 0.1% CHAPS, 5 mM DTT, 2 mM EDTA), followed by 10 μL of Ac-DEVD-pNA (20 mM), bringing the total volume of each well to 100 μL. The effect of Ac-DEVD-CHO, a caspase-3 inhibitor, on caspase-3 activity was studied simultaneously. As per the manufacturer’s instructions, 10 μL of the inhibitor Ac-DEVD-CHO (200 μM) was added to a well containing the assay buffer, followed immediately by the cell lysate and 10 μL of the substrate Ac-DEVD-pNA (2 mM). Caspase-3 activity was assessed by measuring the absorbance released by the pNA molecule at 405 nm using a Bio-Rad Benchmark Plus Microplate Spectrophotometer. Caspase-3 activity was expressed by measuring the ratio of the mean caspase-3 activity of treated cells relative to untreated cells.

#### 3.4.3. DNA Fragmentation by Immunochemical Detection of Histone-Complexed Mono- and Oligonucleosomes

The enrichment of the cytoplasm of treated cells by mono- and oligonucleosomes are used to study whether enhanced DNA fragmentation is induced by diarylpentanoid treatment. This determination was made following the manufacturer’s instructions of the Cell Death Detection ELISA^PLUS^ kit (Roche Diagnostics, Mannheim, Germany). Briefly, HeLa and CaSki cervical cancer cells were exposed to MS17 for 24 and 48 h at concentrations of their respective EC_50_, 2× EC_50_ and 3× EC_50_. Following treatment, the cells were incubated in lysis buffer and centrifuged, after which the supernatant was transferred to streptavidin coated plates and shaken with the provided immunoreagent (mixture of anti-DNA-POD and anti-histone biotin) for 2 h. This incubation period was followed by thorough rinsing with incubation buffer to remove unbound antibody and processed for colour development by addition of the chromogenic substrate for the peroxidase containing 2,2′-azino-di-[3-ethylbenzthiazoline sulfonate (ABTS) and photometric analysis at 405/490 reference wavelengths. Enrichment factor, defined as the release of mono- and oligonucleosomes into the cytoplasm, was calculated using the formula below:

(4) $$\text{Enrichment factor =}\frac{\text{Absorbance of Test }-\text{ Absorbance of Blank (substrate only)}}{\text{Absorbance of Control }-\text{ Absorbance of Blank (substrate only)}}$$

### 3.5. Quantitative Reverse-Transcription Polymerase Chain Reaction (RT-PCR) for Viral Oncogenes E6 and E7

RNA extraction was performed using the Qiagen RNeasy^®^ Mini Kit (Qiagen, Valencia, CA, USA). According to the manufacturer’s instructions, treated HeLa and CaSki cells were lysed using Buffer RLT (supplemented with β-mercaptoethanol) and centrifuged for 3 min at maximum speed. 70% ethanol was added to the lysate and placed in an RNeasy Mini spin column and spun at ≥8000× *g* for 15 s. Flow-through was discarded and the pellet was subjected to a series of wash steps using buffers RW1 and RPE and subsequent RNA elution. On-column RNase-Free DNase (Qiagen) treatment was incorporated into the extraction protocol to eliminate DNase contamination of genomic DNA. Concentration and purity of extracted RNA was measured spectrophotometrically using NanoDrop 1000 (Thermo Fisher Scientific, Wilmington, DE, USA). Two μg of total RNA per 20 μL reaction was used for complementary DNA (cDNA) synthesis using a High Capacity RNA-to-cDNA kit (Applied Biosystems, Life Technologies, Foster City, CA, USA). Appropriate volumes of RNA sample were added to 10 μL 2XRT Buffer and 1 μL 20X RT Enzyme Mix and the total volume was adjusted to 20 μL using nuclease-free water. Reverse transcription was performed on the samples using the following conditions; 37 °C for 60 min and 95 °C for 5 min. The sequences used to design the primers and TaqMan^®^ probes were obtained from previous publications [70,71], synthesized by Applied Biosystems and optimized to amplify the HPV16 and HPV18 E6 and E7 regions (sequences listed in Table 2) using the StepOnePlus™ Real-Time PCR system (Applied Biosystems). The optimized concentrations of the primers and probes used were 900 nm and 250 nm respectively. Human VIC^®^-MGB ACTB (β-actin) was used as an endogenous control for internal normalisation and relative gene expression quantification. Each reaction was run in duplicates and used to calculate relative gene expression using the 2^−∆∆CT^ method. Expression is reported as fold change in the level of mRNA of treated cells relative to untreated cells, normalised to β-actin expression.

**Table 2:** Primer and probe sequences used in quantitative TaqMan^®^ real-time PCR assays measuring the specific viral oncogene expression after MS17 treatment.

Target	Forward Primer	Reverse Primer	Fluorescent Hybridisation Probe ^a^
HPV 16E6	CTGCGACGTGAGGTATATGACTTT	ACATACAGCATATGGATTCCCATCT	6FAM-CTTTTCGGGATTTATGC-MGB-NFQ [71]
HPV 16E7	CAAGTGTGACTCTACGCTTCGG	GTGGCCCATTAACAGGTCTTCCAA	6FAM-TGCGTACAAAGCACACACGTAGACATTCGT-TAMRA [71]
HPV 18E6	CTATAGAGGCCAGTGCCATTCG	TTATACTTGTGTTTCTCTGCGTCG	6FAM-CAACCGAGCACGACAGGAACGACTCCA-TAMRA [70]
HPV 18E7	TAATCATCAACATTTACCAGCCCG	CGTCTGCTGAGCTTTCTACTACTA	6FAM-CGAGCCGAACCACAACGTCACACAATGTT-TAMRA [70]

^a^ FAM™ was used as the reporter dye in all probes; TAMRA™ and MGB-NFQ were used as quencher dyes depending on probe sequence length.

### 3.6. Statistical Analysis

Results were presented as means ± S.E. All samples were measured in triplicates (unless stated otherwise) for each independent experiment. Comparison between datasets was performed using two-way analysis of variance (ANOVA) followed by Dunnet’s multiple group comparison test. Statistical significance between groups was accepted at *p* < 0.05. All statistical analyses were performed using GraphPad Prism version 6.00 for Windows, GraphPad Software (San Diego, CA, USA).

## 4. Conclusions

In conclusion, 1,5-bis(2-hydroxyphenyl)-1,4-pentadiene-3-one (**MS17**) was shown to have significant cytotoxic effects on HeLa and CaSki cervical cancer cells, denoted by its relatively low EC_50_ value and its significant anti-proliferative effects at 48 and 72 hours of treatment compared to the other curcumin analogues tested here. Its apoptosis-inducing ability and down-regulation of HPV-positive oncogenes make it a prospective option for further investigation into its chemotherapeutic potential for cervical cancer treatment.
